# *NR5A1* gene variants: variable phenotypes, new variants, and genotype-phenotype correlations

**DOI:** 10.3389/fendo.2026.1832270

**Published:** 2026-08-13

**Authors:** Lele Li, Di Mao, Xiaoqiao Li, Yanning Song, Xiaoya Ren, Chunxiu Gong

**Affiliations:** Department of Endocrinology, Genetics, Metabolism and Adolescent Medicine, Beijing Children’s Hospital, The Capital Medical University, National Center for Children’s Health, Beijing, China

**Keywords:** disorders of sex development, genotype–phenotype correlation, NR5A1, oligogenic inheritance, pediatric endocrinology

## Abstract

**Introduction:**

*NR5A1* variants are among the most frequent monogenic causes of disorders of sex development (DSD). However, genotype–phenotype correlations remain unclear, and oligogenic contributions to variability are underexplored in non-consanguineous Chinese populations.

**Methods:**

In this single-center, retrospective cohort study, we evaluated clinical, hormonal, and genetic features of a single-center Chinese pediatric cohort with *NR5A1* variants. We assessed genotype–phenotype correlations and intrafamilial variability and performed whole-exome sequencing in a subset to identify additional candidate variants.

**Results:**

In 46,XY patients, gonads were testes in all evaluated cases; three had residual Müllerian structures, and three had primary adrenal insufficiency. The median external masculinization score was 3.0 (2.5–5.0). Among individuals initially assigned female, 73% (33/45) later underwent sex reassignment to male. Follicle-stimulating hormone levels increased significantly during puberty. We identified 76 *NR5A1* variants, most commonly in the hinge region (37/76), followed by the DNA-binding (28/76) and ligand-binding (7/76) domains. Intrafamilial phenotypes varied markedly, including primary ovarian insufficiency or irregular menstruation in women and oligospermia or DSD in men. The external masculinization score and penile length standard deviation score did not differ between missense and loss-of-function variants (median score −3.63 vs −4.71; all *P* > 0.05) and remained similar across functional domains; testis location scores and sex assignment were also comparable. Whole-exome sequencing in 25 patients identified 49 potentially deleterious candidate variants in 36 additional genes.

**Conclusions:**

*NR5A1*-related DSD shows substantial phenotypic and intrafamilial variability without consistent genotype–phenotype correlations. Concomitant variants in other DSD-related genes support an oligogenic contribution to disease expression.

## 1 Introduction

Disorders/differences in sex development (DSD) comprise a broad, heterogeneous group of congenital conditions characterized by atypical development of genetic, gonadal, or phenotypic sex, with abnormal internal and/or external genitalia. Monogenic DSD etiologies include pathogenic variants in genes governing gonadal and genital duct development, gonadal/adrenal steroidogenesis, and target-cell responsiveness. Heterozygous pathogenic *NR5A1* variants account for ~10–20% of 46,XY DSD (1).

*NR5A1* (nuclear receptor subfamily 5 group A member 1) maps to 9q33.3 and spans >30 kb, comprising one noncoding exon (exon 1) and six coding exons (exons 2–7) (2). The encoded steroidogenic factor 1 (SF-1) contains a zinc-finger DNA-binding domain (DBD), a ligand-binding domain (LBD), activation domains (AF-1/AF-2), an accessory region, and a flexible hinge region. The DBD harbors two Cys4 zinc-finger motifs and a conserved Ftz-F1 box implicated in DNA interaction, whereas the hinge region stabilizes the LBD and mediates protein–protein interactions regulating SF-1 transcriptional activity (3, 4).

In humans, *NR5A1* variants cause a wide phenotypic spectrum. Initial reports described 46,XY DSD with adrenal insufficiency and a 46,XX girl with adrenal insufficiency (5). Subsequent studies extended the phenotype to 46,XY DSD (from complete/partial gonadal dysgenesis to isolated micropenis without adrenal insufficiency), 46,XY ovotesticular DSD, 46,XX primary ovarian insufficiency (POI), and 46,XX testicular/ovotesticular DSD (6–10). *NR5A1* variants have also been identified in men with unexplained infertility and in some patients with spleen anomalies, adrenal tumors, or endometriosis (11–14). Overall, clinical expressivity is highly variable.

Phenotypic variability occurs across different *NR5A1* variants and among carriers of the same variant, including within families, and robust genotype–phenotype correlations remain elusive. Oligogenic inheritance has been proposed, suggesting that additional variants within sex-development pathways may modulate *NR5A1*-associated phenotypes (15). Here, we report clinical features, hormonal profiles, and the molecular landscape of *NR5A1* variants in a single-center Chinese pediatric cohort. We retrospectively characterized individual- and family-level phenotypes and genotypes of individuals and families with *NR5A1/SF-1* variants using existing data, with emphasis on detailed DSD phenotyping and intrafamilial variability. These results refine our understanding of *NR5A1*-related heterogeneity and may inform diagnostic and management strategies.

## 2 Materials and methods

This study was approved by the Ethics Committee of the Beijing Children’s Hospital, Capital Medical University (Ethics Approval Number 2018-209). Written informed consent was obtained from all patients or their legal guardians. This study was conducted in accordance with the principles of the Declaration of Helsinki.

### 2.1 Patients

Patients aged 2 months–17 years with ambiguous external genitalia and *NR5A1* variants were recruited from 87 unrelated families. Clinical diagnosis for patients with *NR5A1* variants was based on: (1) external genital undervirilization features (e.g., hypospadias, microphallus, cryptorchidism, clitoromegaly, or complete female external genitalia) and/or adrenal insufficiency; and (2) an *NR5A1* variant classified as pathogenic, likely pathogenic, or a variant of uncertain significance according to the American College of Medical Genetics and Genomics and the Association for Molecular Pathology (ACMG/AMP) guidelines (16). We excluded patients with 46,XX disorders of androgen excess, disorders of Müllerian development, or cloacal exstrophy, and patients with 46,XY DSD due to Leydig cell defects, persistent Müllerian duct syndrome, 5α-reductase type 2 deficiency, androgen insensitivity syndrome, or cloacal exstrophy, as these conditions are caused by other specific genetic defects. Patients with insufficient mandatory data (e.g., precise *NR5A1* variant annotation at the DNA and protein levels or karyotype) were also excluded.

### 2.2 Clinical information

Clinical data were extracted from medical records. Two experienced pediatric endocrinologists performed physical examinations and assessments. Variables included age at visit, sex, chief complaint, family history, anthropometrics (including birth length, birth weight, gestational age, and gestational history), genital findings (clitoral/penile length, testis size, testis position, urethral meatus location, and scrotal appearance), facial features, liver and kidney function, and electrolyte levels. Pituitary hormones and testosterone (T) concentrations were measured. B-mode ultrasound or magnetic resonance imaging was used to assess the kidneys, adrenal glands, pelvic gonads, and reproductive ducts.

Phenotypes at the first clinic visit were classified using the external masculinization score (EMS), sub-scores for urethral meatus and gonad location, and the penile length standard deviation score (PL-SDS). Stretched penile length was measured as previously described (17) and compared with reference values from China (18). Micropenis was defined as stretched penile length < −2.5 standard deviations for age, according to the 2023 Expert Consensus for the Management of Congenital Micropenis (19).

Hormonal measurements included basal luteinizing hormone (LH), follicle-stimulating hormone (FSH), estradiol (E2), T, and dihydrotestosterone (DHT). Levels of LH, FSH, E2, and T were measured using chemiluminescent immunoassay using an Immulite 2000 (Siemens Healthcare, Sudbury, UK). DHT was measured using liquid chromatography coupled with mass spectrometry (1200 Series HPLC system, Agilent, Santa Clara, CA; API5000 tandem mass spectrometer, AB Sciex Pte, Framingham, MA, USA). Stimulated T and DHT levels were measured following the human chorionic gonadotropin (hCG) standard, and prolonged tests were performed as previously described by Wang et al. (20). Adrenocorticotropic hormone (ACTH) and cortisol were evaluated using the radioimmunoassay method (MAGLUMI^®^2000). Anti-Müllerian hormone (AMH) and inhibin B levels were evaluated using electrochemiluminescence and enzyme-linked immunosorbent assays, respectively.

### 2.3 Sex assignment and follow-Up

All patients underwent comprehensive assessment by a multidisciplinary team including an endocrinologist, urologist, and psychologist. This assessment incorporated external genital phenotype, response to androgen treatment, and psychosexual factors (e.g., interests, behaviors, social preferences, and personality). Recommendations regarding sex reassignment were made with careful consideration of both parental and patient preferences. During follow-up, patients underwent physical examination, hormone testing, and psychological questionnaires every 3–6 months.

### 2.4 Molecular analysis

Karyotyping was performed for all patients. Genetic testing consisted of targeted Sanger sequencing of four DSD-related genes (*SRD5A2, AR, NR5A1*, and *SRY*) and next-generation sequencing of a panel of 127 DSD-related genes, or whole-exome sequencing (WES), depending on the clinical features and financial resources of the patients. Next-generation sequencing was performed by qualified domestic companies (MyGenostics, Beijing Kangso Medical Laboratory, and the Chigene Translational Medical Research Center). Candidate variants were confirmed by Sanger sequencing, and parental validation was performed when parental samples were available. Genomic data were compared with the NCBI reference sequence NM_004959. Pathogenic evaluation of the variants was based on the ACMG/AMP guidelines (20).

### 2.5 Genotype–phenotype correlation and pedigree analysis

The following subgroups were compared for differences in patients’ EMS, gonadal location, PL-SDS, and assigned sex: variants located in different domains (DBD, LBD, or hinge region) and variants of various types (missense vs. loss-of-function [LoF]). LoF variants comprise several variant forms, including nonsense, frameshift, and splicing. Medical reports were used to extract family histories of gonadal anomalies, infertility, POI, irregular menstruation, and oligospermia. Sanger sequencing of the *NR5A1* gene was conducted on available family members.

### 2.6 Oligogenic etiology analysis

WES data were filtered using a disease-tailored list of known *NR5A1*-related and DSD-related candidate genes (N = 606), similar to the algorithm previously designed by Camats et al. (15). A project-specific filter for DSD- and *NR5A1*-related genes was generated by searching published literature and databases. The following bioinformatics software tools were used to interpret and classify the variants: InterVar (http://wintervar.wglab.org/; clinical interpretation of genetic variants using the ACMG/AMP 2015 guidelines), VarSome (The Human Genomics Search Engine; https://varsome.com/), ClinVar (https://www.ncbi.nlm.nih.gov/clinvar/), and Alamut Visual 2.11 (https://www.interactive-biosoftware.com/es/alamut-visual/). Following genetic annotation, variant filtering and analysis were performed via a stepwise strategy. First, whole-exome sequencing (WES) data were screened against a curated list of known and candidate genes associated with NR5A1 dysfunction and DSD pathogenesis. Second, rare variants with a minor allele frequency (MAF) < 0.05, as well as variants absent from the gnomAD, 1000 Genomes (Chinese population), and ExAC (East Asian) databases, were retained for subsequent evaluation. Third, variants with limited research significance were excluded according to the following criteria: (1) variants detected in more than two enrolled patients; (2) variants located in repetitive genomic regions; (3) variants in highly polymorphic gene regions with MAF > 0.05; and (4) variants with low sequencing coverage or poor variant quality.

### 2.7 Statistical analysis

Descriptive analysis included the median with interquartile ranges (25th percentile P25 and 75th percentile P75) for continuous variables, and frequencies and percentages for categorical variables. Non-parametric data were analyzed using the Mann–Whitney *U* test. Differences between the proportions of qualitative data were assessed using the χ2 test. Statistical significance was defined as P < 0.05. Bonferroni correction was performed for multiple pairwise comparisons among the three groups, which yielded an adjusted significance cutoff of P < 0.0167. We used SPSS v.25.0 (IBM Corp., Armonk, NY, USA) statistical software for data cleaning and analyses, and GraphPad Prism (version 9.5.1) (GraphPad Software, San Diego, CA, USA) for constructing graphs.

## 3 Results

### 3.1 Clinical features

We enrolled 87 pediatric patients carrying *NR5A1* variants from a larger single-center cohort in China (85 patients with the 46,XY karyotype and two with the 46,XX karyotype). Detailed clinical information for each patient is presented in **Supplementary Table 1**. The results for 30 of these patients have been previously published (21). Of the two patients with 46,XX, one had ovotestis DSD, and the other had primary adrenal insufficiency without DSD. The median age of the patients was 1.5 years, with an interquartile range of 0.92–6.62 years. In 46,XY patients with available gonadal evaluation/tissue, gonads were testes, with three patients having residual Müllerian structures and three others with primary adrenal insufficiency. At the initial visit, 28 individuals were in mini-puberty, 44 were in pre-puberty, and 14 were in puberty (**Table 1**). Upon analysis of patients’ data who had complete EMS records (n=75), the median value of this parameter was 3.0 (2.5, 5.0). The median PL-SDS was −3.83 (−5.42, −2.30) (n=66). We classified all individuals into three groups based on the severity of the DSD phenotype. According to this classification, 29 (38.7%), 34 (45.3%), and 12 (16.0%) patients had severe, moderate, and mild DSD phenotypes, respectively.

**Table 1:** Clinical information and hormone profiles in the full analysis set.

Indicator	Overall (n=86)	DBD (n=28)	Hinge region (n=7)	LBD (n=37)
Age at first visit,years				
Median (IQR)	1.5 (0.92,6.62)	1.71 (0.77,5.12)	1.25 (0.42,6.17)	1.5 (0.96,9.71)
mini-puberty, n(%)	28 (32.6%)	11 (39.2%)	2 (28.6%)	12 (32.4%)
prepuberty, n(%)	44 (51.2%)	14 (50.0%)	5 (71.4%)	16 (43.2%)
puberty, n(%)	14 (16.3%)	3 (10.7%)	0 (0%)	9 (24.3%)
Registered gender at the initial visit				
Male, n(%)	42 (48.8%)	14 (50.0%)	4 (57.1%)	19 (51.3%)
Female, n(%)	44 (51.2%)	14 (50.0%)	3 (42.9%)	18 (48.7%)
Gender at last follow-up				
Male, n(%)	74 (86.0%)	23 (82.1%)	5 (71.4%)	35 (94.6%)
Female, n(%)	6 (7.0%)	3 (10.7%)	1 (14.3%)	1 (2.7%)
Undetermained, n(%)	6 (7.0%)	2 (7.2%)	1 (14.3%)	1 (2.7%)
Phenotypic characteristics				
EMS^**^Median(IQR)	3.0 (2.5,5) (n=75)	2.5 (1,6.75) (n=24)	3 (2.5,4.5) (n=5)	3.0 (2.5,5.25) (n=33)
EMS ≤ 3 (severe), n(%)	29 (38.7%)	13 (54.2%)	1 (20.0%)	9 (27.2%)
EMS>3,≤6 (moderate), n(%)	34 (45.3%)	5 (20.8%)	4 (80.0%)	20 (60.6%)
EMS>6 (mild), n(%)	12 (16.0%)	6 (25.0%)	0	4 (12.2%)
Urethral meatus score, Median(IQR)	0.0 (0.0,0.0) (n=75)	0.0 (0.0,0.0) (n=24)	0.0 (0.0,0.0) (n=5)	0.0 (0.0,0.0) (n=33)
Gonad position score, Median(IQR)	2.5 (1.5,3.0) (n=75)	2.0 (1.0,3.0) (n=24)	3.0 (2.5,3.0) (n=5)	3.0 (1.75,3.0) (n=33)
PL-SDS, Median(IQR)	-3.83 (-5.42,-2.30) (n=66)	-5.29 (-6.79,-2.265) (n=21)	-4.71 (-6.42,-1.83) (n=5)	-3.29 (-3.86,-1.96) (n=28)#
LH (IU/L)				
mini-puberty	1.5 (0.68,2.84) (n=28)	0.68 (0.27,2.87) (n=11)#	2.12,18.4 (n=2)$	1.33 (0.80,2.60) (n=12)
prepuberty	0.18 (0.10,0.33) (n=44)&	0.17 (0.10,0.44) (n=13)	0.34 (0.14,0.63) (n=5)	0.20 (0.13,0.29) (n=15)
puberty	4.12 (3.76,15.27) (n=14)&*	6.51 (3.81,18.50) (n=3)	ND (n=0)	4.25 (3.88,16.70) (n=9)
FSH (IU/L)				
mini-puberty	9.58 (5.33,17.5) (n=28)	9.28 (5.54,21.80) (n=11)	11.84,24.3 (n=2)	8.41 (2.57,16.40) (n=12)
prepuberty	3.15 (2.08,4.71) (n=42)&	3.52 (2.49,4.96) (n=13)	3.01 (2.09,4.80) (n=5)	3.18 (1.73,4.34) (n=15)
puberty	28.68 (18.13,44.60) (n=14)&*	28.96 (31.9,51.50) (n=3)	ND (n=0)	28.40 (22.40,54.65) (n=9)
LH/FSH ratio				
mini-puberty	0.16 (0.84,0.25) (n=28)	0.08 (0.02,0.16) (n=11)	0.18,0.75 (n=2)	0.17 (0.11,0.31) (n=12)
prepuberty	0.06 (0.04,0.25) (n=42)&	0.05 (0.04,0.12) (n=13)	0.07 (0.05,0.28) (n=5)	0.06 (0.05,0.13) (n=15)
puberty	0.24 (0.13,0.43) (n=14)	0.20 (0.13,0.35) (n=3)	ND (n=0)	0.19 (0.13,0.38) (n=9)
T (ng/dl)				
mini-puberty	35.1 (20.0,141.0) (n=27)	22.20 (15.61,50.80) (n=11)	116.1,314 (n=2)#	49.0 (22.47,208.0) (n=11)
prepuberty	20.00 (20.00,20.00) (n=41)&	20.00 (15.98,20.00) (n=13)	20.0 (20.0,20.0) (n=5)	20.0 (20.0,20.0) (n=15)
puberty	169.50 (52.67,275.91) (n=14)&*	53.3 (20,271.55) (n=3)	ND (n=0)	130.0 (75.40,271.0) (n=9)
DHT (pg/ml)				
mini-puberty	121.7 (50.0,232.6) (n=12)	50.0 (50.0,163.6) (n=4)	98.28 (n=1)	207.3 (112.75,431.95) (n=6)
prepuberty	50.00 (27.44,108.0) (n=15)&	90.35 (53.45,148.77) (n=4)	26.22,25.0 (n=2)	79.70 (37.5,128.50) (n=5)
puberty	170.15 (140.82,294.60) (n=6)&*	ND (n=0)	ND (n=0)	188.30 (139.55,316.20) (n=5)
T after hCG stimulation test (ng/dl)				
prepuberty	157.0 (24.87,329.14) (n=44)	181.98 (41.95,442.60) (n=10)	157.0 (74.65,281.50) (n=5)	203.50 (117.50,378.00) (n=14)
DHT after hCG stimulation test (pg/ml)				
prepuberty	226.3 (86.55,334.00) (n=24)	282.20 (121.17,515.75) (n=6)	236.30 (43.50,478.30) (n=4)	231.20 (139.70,410.20) (n=9)
AMH (ng/dl)				
mini-puberty	23 (8.14,23.0) (n=18)	23 (6.82,23.0) (n=7)	8.59 (n=1)	23.0 (14.0,23.0) (n=8)
prepuberty	21.29 (10.96,23.0) (n=35)	16.68 (10.39,22.57) (n=12)	21.26 (9.45,23.0) (n=4)	23.0 (21.44,23.0) (n=12)
puberty	0.75 (0.23,2.85) (n=10)&*	0.19,0.91 (n=2)	ND (n=0)	0.59 (0.25,2.70) (n=7)
Inhibin B (pg/ml)				
mini-puberty	88.0 (20.84,155.71) (n=17)	99.5 (31.27,138.4) (n=6)	21.5 (n=1)	148.36 (45.87,194.88) (n=8)
prepuberty	70.85 (39.30,125.25) (n=35)	74.7 (42.51,113.79) (n=12)	91.62 (53.98,143.77) (n=4)	83.41 (59.27,205.83) (n=12)
puberty	16.38 (11.02,80.78) (n=10)	7.65,30.40 (n=2)	ND (n=0)	13.57 (11.16,9.57) (n=7)
ACTH (ng/dl)	30.70 (21.15,47.75) (n=73)	27.4 (19.50,68.10) (n=23)	24.60 (16.95,47.20) (n=5)	33.90 (24.60,47.97) (n=32)
Cortisol (pg/ml)	9.44 (9.44,12.35) (n=72)	9.87 (7.88,13.30) (n=23)	8.43 (7.98,9.99) (n=5)	9.31 (6.98,12.70) (n=31)

^#^Compared with the DBD group, *P*<0.05; ^$^Compared with the LBD group, *P*<0.05; ^&^Compared with the mini-puberty group, *P*<0.05; *Compared with the pre-puberty group, *P*<0.05. DBD, DNA-binding domain; LBD, ligand-binding domain; IQR, interquartile range; EMS, external masculinization scores; PL-SDS, penile length standard deviation score; LH, luteinizing hormone; FSH, follicle-stimulating hormone; T, testosterone; DHT, dihydrotestosterone; hCG, human chorionic gonadotropin; AMH, anti-Müllerian hormone; ACTH, adrenocorticotropic hormone; ND, no available data.

**Figure 1:**
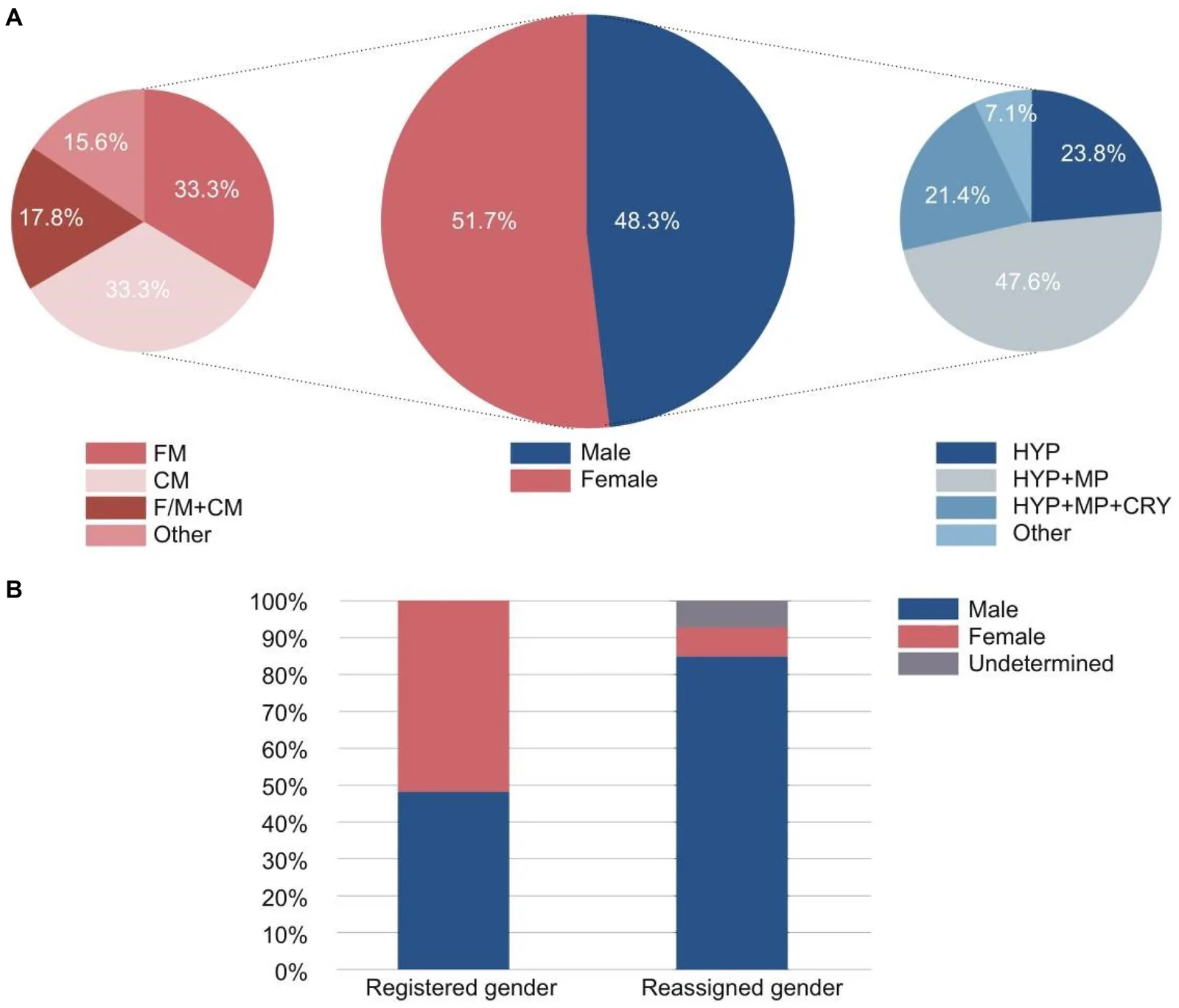
Clinical features of the enrolled *NR5A1*-DSD patients. **(A)** The reason for consultation in different social gender patients at first visit. **(B)** The patients’ social gender at first visit and last visit. FM, fulling or mass at groin or labia; CM, clitoromegaly; MP, micropenis; HYP, hypospadias; CRY, cryptorchidism.

At the initial visit, 42 patients with predominantly male external genitalia were registered as male, including 10 (23.8%) patients with isolated hypospadias, 20 (47.6%) with hypospadias plus micropenis, and nine (21.4%) with hypospadias plus micropenis plus cryptorchidism. In contrast, 45 patients with predominantly female external genitalia were registered as female, including 15 (33.3%) with clitoromegaly, 15 (33.3%) with masses in the groin or labia, and eight (17.8%) with both clitoromegaly and masses in the groin or labia. Further details are presented in **Figure 1A**. After diagnosis, 32 female patients were reassigned to male sex, and none of the male patients were reassigned to female sex. At the last follow-up, the sex was male in 74 patients, female in six patients, and undetermined in six patients (**Figure 1B**).

The hormone measurement results are presented in **Table 1**. The median ACTH level was 30.70 (21.15, 47.75) pg/mL, and the median cortisol level was 9.44 (9.44, 12.35) µg/dL. FSH levels increased significantly during puberty. AMH and inhibin B levels were lower during the pubertal period than during the prepubertal period.

### 3.2 Genetic characteristics

**Figure 2:**
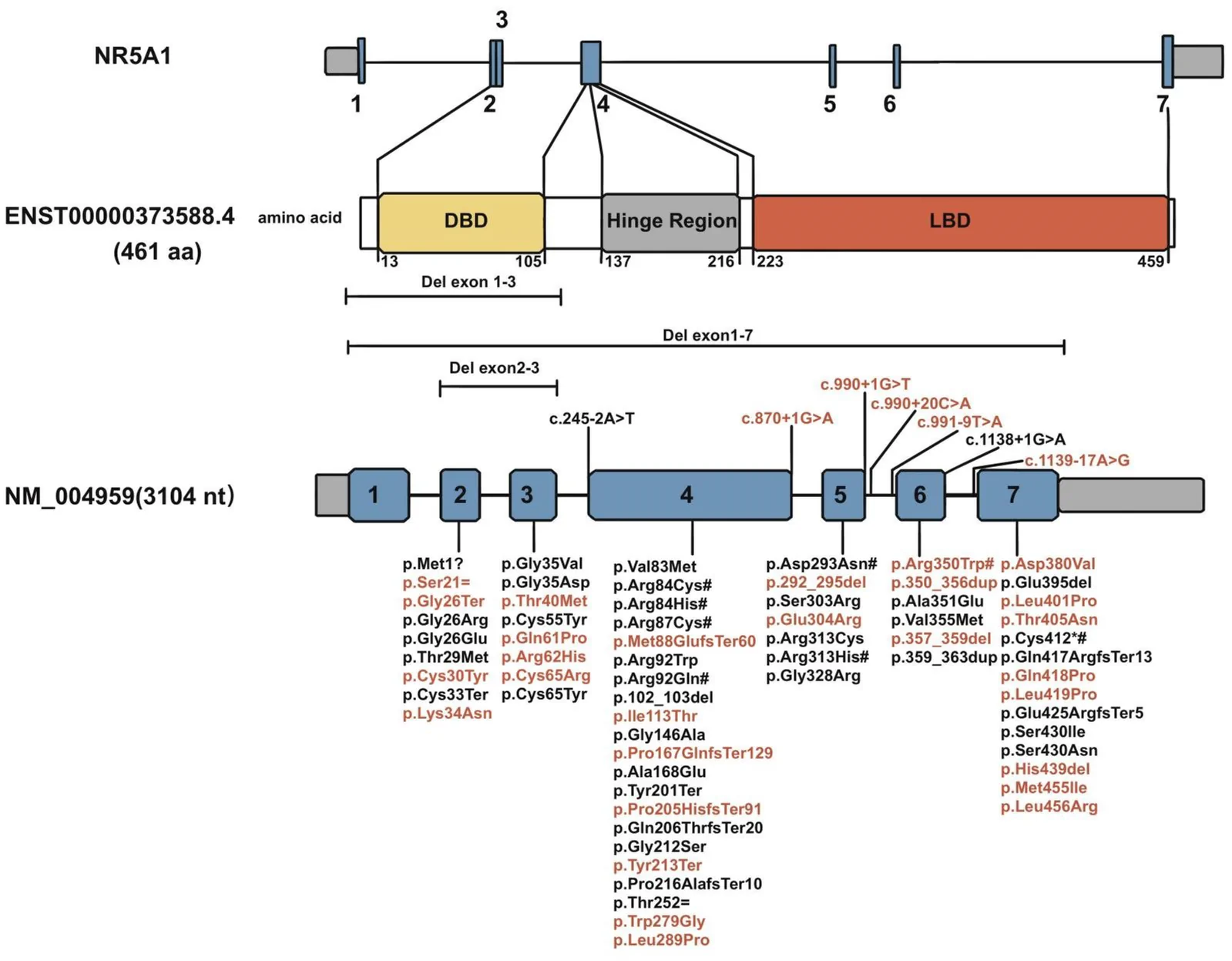
Schematic representation of variant localization of the patients. The *NR5A1* gene transcript was NM_004959. DBD, DNA binding domain; LBD, Ligand binding domain. Red font refers to the presence of novel variants, while # represents recurring variants. Among the recurring variants, the following were observed: c.937C>T:p.Arg313Cys (n=4), c.251G>A:p.Arg84His (n=3), c.250C>T:p.Arg84Cys (n=2), c.259C>T:p.Arg87Cys (n=2), c.275G>A:p.Arg92Gln (n=2), c.877G>A:p.Asp293Asn (n=2), c.1048C>T:p.Arg350Trp (n=2), c.1236C>A:p.Cys412* (n=2).

**Supplementary Table 1** and **Figure 2** show the identified *NR5A1* gene variants. All individuals were heterozygous for *NR5A1* variants. There were 76 variants in 87 patients, including 41 missense, six nonsense, seven frameshift, eight in-frame (two insertions, five deletions, and one deletion-insertion), seven splicing, two synonymous, one start codon, and four gross deletions. Of these, 40 were previously reported, and 36 were novel *NR5A1* variants. Furthermore, seven, 37, and 28 variants were identified in the LBD, hinge region, and DBD, respectively. Exon 4 was the most commonly affected exon, with 32% (28/87) of the patients affected, followed by exon 7 (18%, 16/87).

Approximately 78% (68/87) of the variants were unique and appeared only once. However, several recurring variants were observed multiple times (**Figure 2**). Among the recurring variants, the following were observed: c.937C>T:p. Arg313Cys (n=4), c.251G>A:p.Arg84His (n=3), c.250C>T:p.Arg84Cys (n=2), c.259C>T:p.Arg87Cys (n=2), c.275G>A:p.Arg92Gln (n=2), c.877G>A:p.Asp293Asn (n=2), c.1048C>T:p.Arg350Trp (n=2), c.1236C>A:p.Cys412* (n=2).

### 3.3 Pedigree analysis

In our single-center cohort, parental validation was performed in 78 cases. Of these, 29 patients (37%) inherited the variant from their mothers, whereas 20 (25%) inherited the variant from their fathers. The remaining 29 patients (37%) had *de novo* variants. Among the 49 individuals who inherited variants from their parents, 20% (10/49) were identified as having recurrent variants in this study. The following were observed: c.937C>T:p. Arg313Cys (n=2), c.251G>A:p.Arg84His (n=2), c.250C>T:p.Arg84Cys (n=2), c.877G>A:p.Asp293Asn (n=2), c.1048C>T:p.Arg350Trp (n=2).

**Figure 3:**
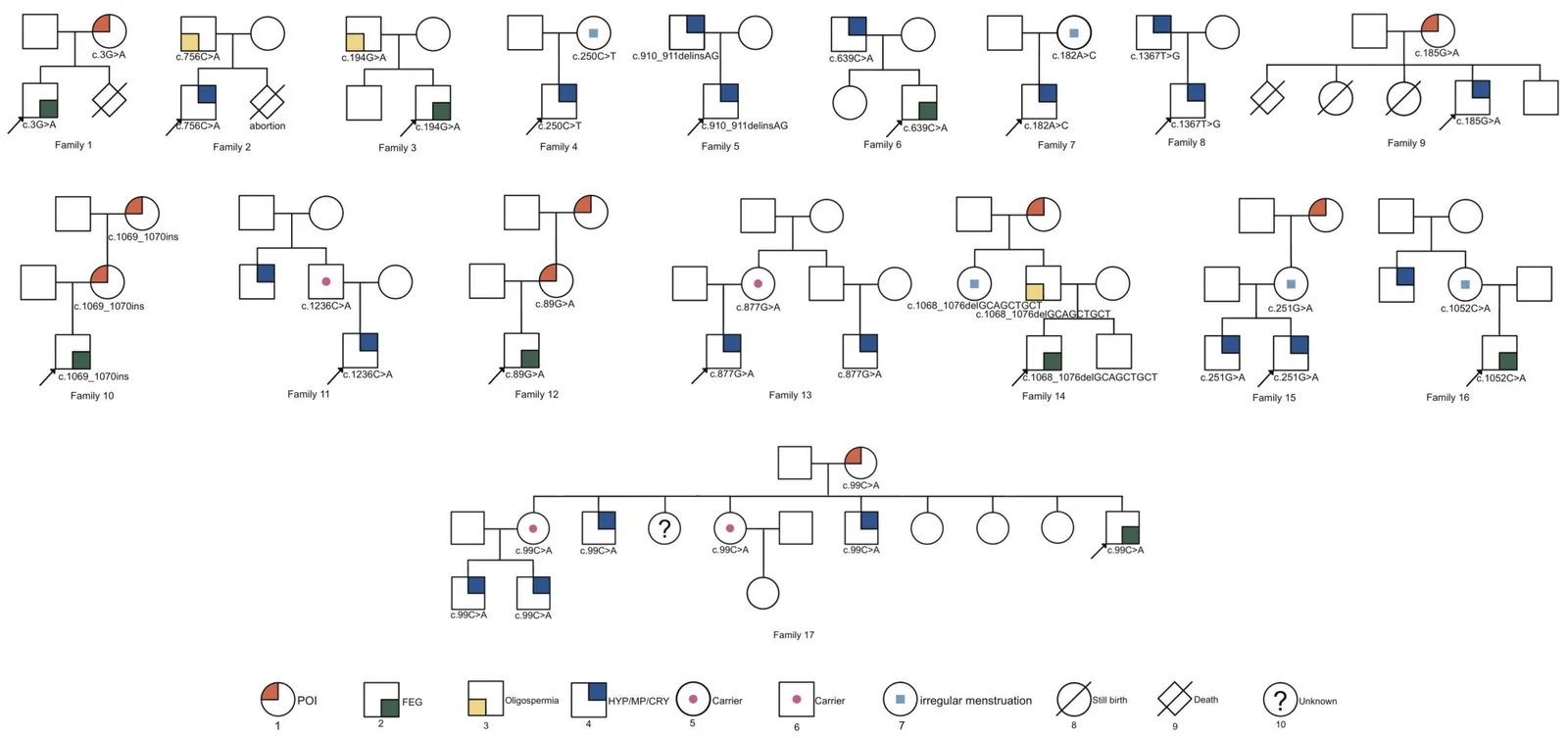
Partial pedigrees of the families. POI, primary ovarian insufficiency; FEG, female external genitalia.

In 49 families (63%), data were obtained from at least one relative with the same *NR5A1* variant as that of the proband. For these 49 families, we again investigated family history in more detail and found that 17 families had a positive family history. The intrafamilial DSD phenotype in these 17 families varied significantly, and the clinical spectrum was wide. Ten cases had variations inherited from their mother, five cases had POI in the mother, four cases had irregular menstruation, and one case had a mother carrying the variation without phenotype, three cases had premature ovarian failure in the maternal grandmother. Seven probands with variations inherited from their father, including three cases of oligospermia and asthenospermia in the father, three cases of DSD phenotype, and one case of a father carrying a variant without phenotype (**Figure 3**).

### 3.4 Genotype–phenotype correlation

**Figure 4:**
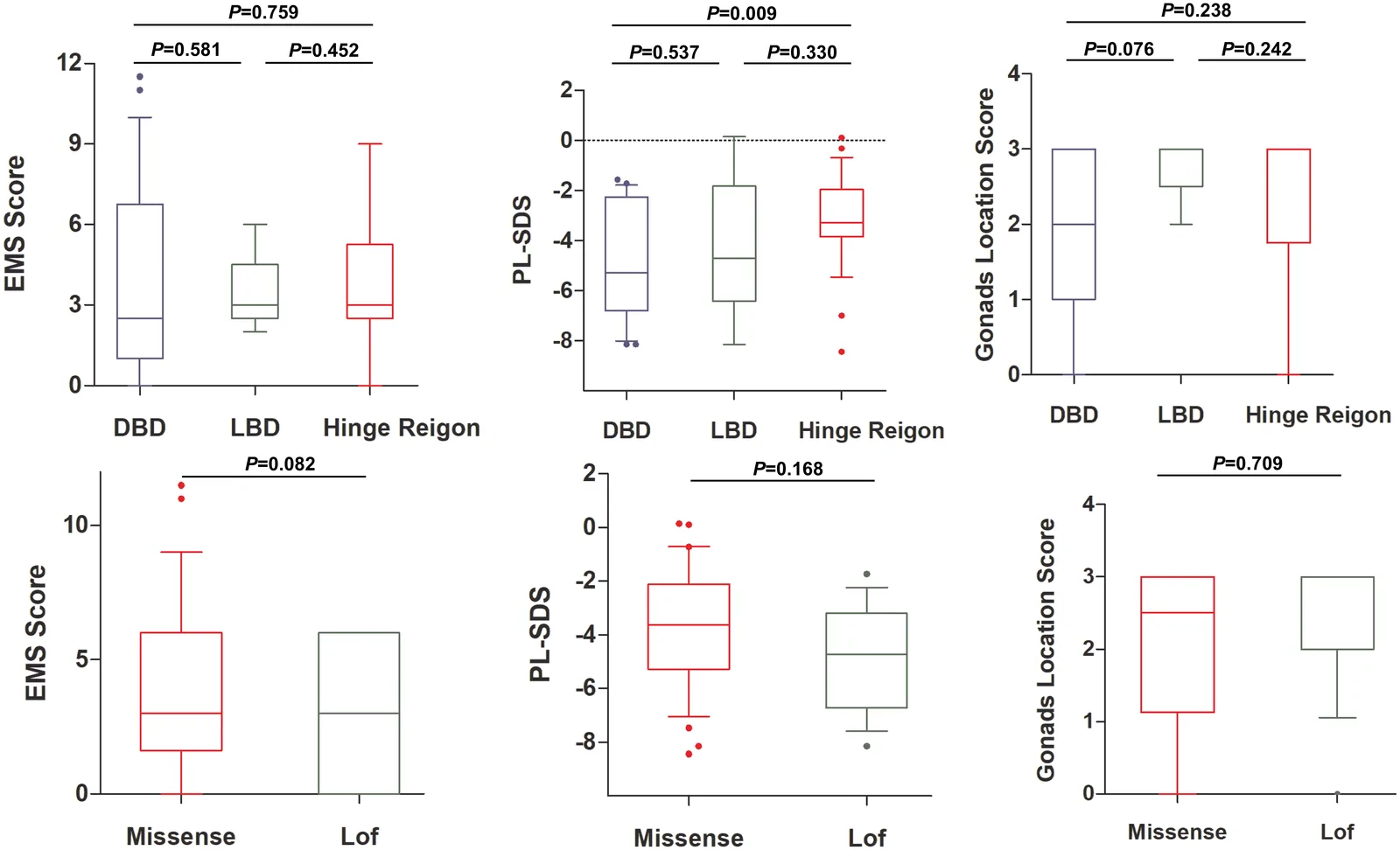
Comparison of phenotypes across NR5A1 genotypes. DBD, DNA binding domain; LBD, Ligand binding domain; PL-SDS, penile length standard deviation score; EMS, external masculinization scores; Lof, loss-of-function.

We classified patients with *NR5A1* variants into missense and LoF groups based on the type of *NR5A1* variant. The EMS and PL-SDS for the missense group were 3 (1.625, 6.0) and −3.63 (−5.29, −2.105), respectively, which were not significantly different from those in the LoF group 3 (2, 3), −4.71 (−6.71, −3.19) (*P* = 0.082 for EMS and *P* = 0.168 for PL-SDS). Further analysis of phenotypic differences among the functional domains showed no statistically significant differences in EMS, testis location scores, PL-SDS, or sex assignment among the three groups (all *P*>0.05). The results are shown in **Figure 4**.

We compared phenotypic differences in patients with repetitive variants. Patients with c.937C>T, p. Arg313Cys (n=4), c.251G>A:p.Arg84His (n=3), c.250C>T:p.Arg84Cys (n=2), c.259C>T:p.Arg87Cys (n=2), c.275G>A:p.Arg92Gln (n=2), c.877G>A:p.Asp293Asn (n=2), c.1048C>T:p.Arg350Trp (n=2), c.1236C>A:p.Cys412* (n=2) (**Supplementary Table 1**). The results showed that the clinical phenotypes varied even among patients carrying the same *NR5A1* variant, indicating the absence of any specific genotype–phenotype correlation.

### 3.5 Identification of an oligogenic etiology

**Figure 5:**
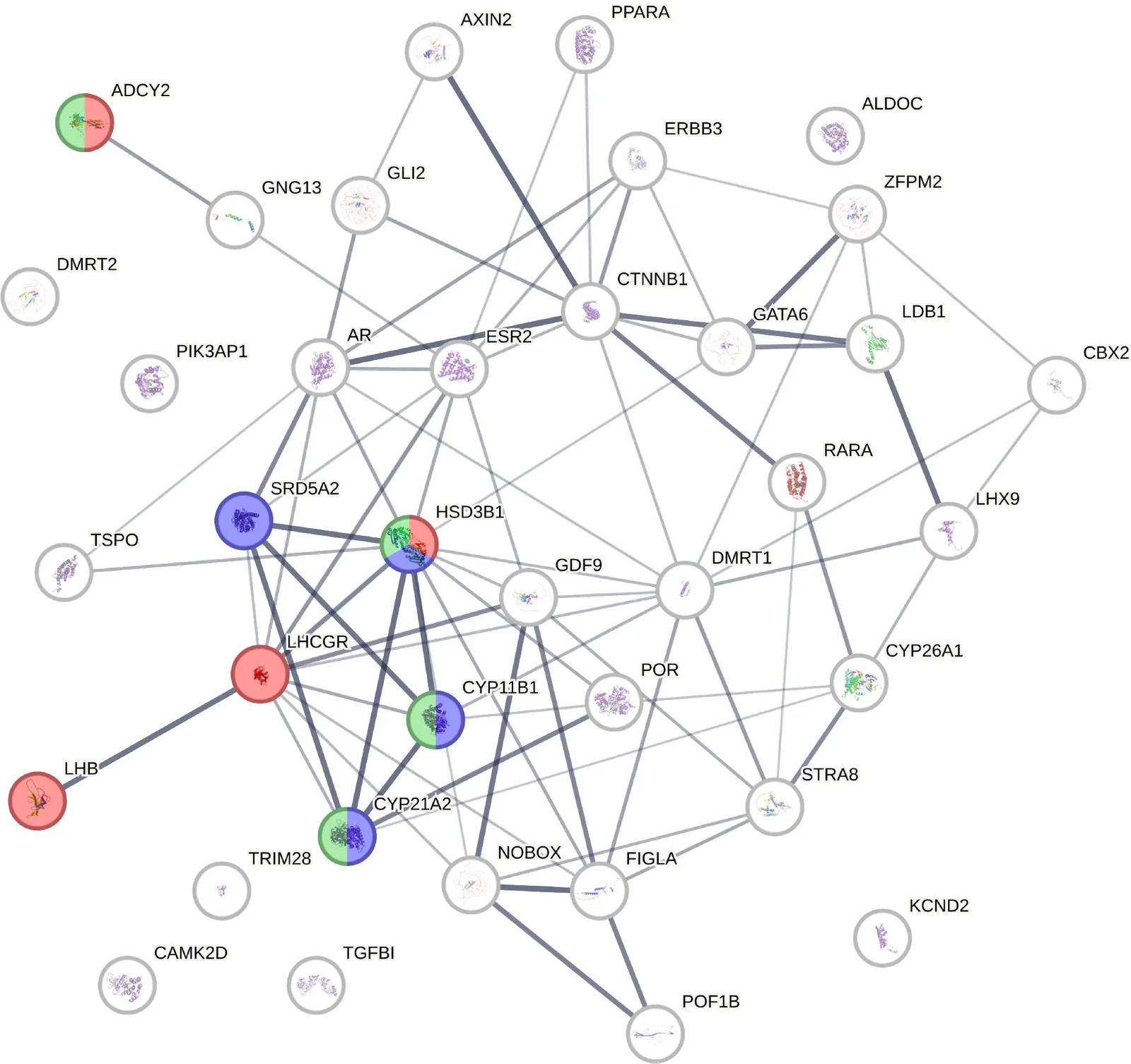
Interaction network of DSD- and *NR5A1*-related genes identified in DSD patients.

Information on the genetic testing used for the identification of *NR5A1* variants was available for 85 individuals. Of these, 25 were tested using WES. WES data were filtered using common tools and disease customization algorithms, including known and candidate *NR5A1*- and DSD-related genes. Forty-nine potentially deleterious/candidate variants in 36 genes (*ADCY2*, *ALDOC*, *AR*, *CAMK2D*, *CBX2*, *CTNNB1*, *CYP11B1*, *CYP21A2*, *CYP26A1*, *DMRT1*, *DMRT2*, *ERBB3*, *ESR2*, *FIGLA*, *GATA6*, *GDF9*, *GLI2*, *GNG13*, *HSD3B1*, *KCND2*, *LDB1*, *LHB*, *LHCGR*, *LHX9*, *NOBOX*, *PIK3AP1*, *POF1B*, *POR*, *PPARA*, *RARA*, *SRD5A2*, *STRA8*, *TGFBI*, *TRIM28*, *TSPO*, and *ZFPM2*) were identified, which may contribute to each patient’s phenotype (shown in **Table 2**). *ADCY2* was the most commonly identified gene. We performed interactome analysis to identify DSD-related genes using bioinformatics software to assess possible gene-protein interactions. Interactome analysis revealed a functional association network among the 36 identified gene products (shown in **Figure 5**). While not all proteins interact directly, they converge on several key signaling hubs critical for gonadal development and steroidogenesis, including the WNT/β-catenin pathway (CTNNB1), androgen signaling (AR/ESR2), and TGF-β signaling (TGFBI). This suggests that the candidate variants may converge on common biological pathways that modulate the core NR5A1-related phenotype.

**Table 2:** Oligogenic mutations of 25 patients.

Case	Number of gene	Number of mutant alleles	Gene	Nucleotide change	Amino acid change	Mutation type	Region	ACMG
2	0	0	ND	ND	ND	ND	ND	ND
32	2	3	*ZFPM2*	c.2287G>A	p.Val763Ile	mis	exon8	VUS
			*RARA*	c.13A>T	p.Ser5Cys	mis	exon2	VUS
			*RARA*	c.1013-6C>T	ND	Splicing near	IVS7	LB
33	5	5	*FIGLA*	c.11C>A	p.Ala4Glu	mis	exon1	VUS
			*LHCGR*	c.1875A>G	p.Ile625Met	mis	exon11	VUS
			*ALDOC*	c.1000-18C>G	ND	Splicing near	IVS8	VUS
			*TRIM28*	c.1217-15C>T	ND	Splicing near	IVS8	VUS
			*ERBB3*	c.2056-5_2056-3delCTC	ND	ND	IVS17	VUS
34	2	2	*TGFBI*	c.1405C>T	p.Arg469Cys	mis	exon10	VUS
			*STRA8*	c.238C>A	p.Gln80Lys	mis	exon3	VUS
35	1	1	*ADCY2*	c.211-3C>T	ND	Splicing near	IVS1	LB
36	5	5	*GLI2*	c.1577C>G	p.Ala526Gly	mis	exon11	VUS
			*ADCY2*	c.2036G>A	p.Arg679Gln	mis	exon16	VUS
			*KCND2*	c.1278 + 4C>T	ND	Splicing near	IVS2	VUS
			*LHB*	c.262C>T	p.Arg88Trp	mis	exon3	VUS
			*ERBB3*	c.3380G>A	p.Arg1127His	mis	exon27	VUS
39	3	3	*GDF9*	c.770C>T	p.Thr257Ile	mis	exon2	VUS
			*CYP26A1*	c.705 + 3A>G	ND	Splicing near	IVS3	VUS
			*PPARA*	c.370-13A>G	ND	Splicing near	IVS5	VUS
46	3	3	*GLI2*	c.4160C>T	p.Ala1387Val	mis	exon14	VUS
			*STRA8*	c.238C>A	p.Gln80Lys	mis	exon3	VUS
			*ADCY2*	c.2776-11T>C	ND	Splicing near	IVS21	VUS
51	3	3	*ADCY2*	c.211-3C>T	ND	Splicing near	IVS1	LB
			*AR*	c.170T>A	p.Leu57Gln	mis	exon1	VUS
			*CYP11B1*	c.1502C>G	p.Ala501Gly	mis	exon9	VUS
58	2	2	*ADCY2*	c.211-3C>T	ND	Splicing near	IVS1	LB
			*STRA8*	c.877G>C	p.Glu293Gln	mis	exon6	VUS
59	1	1	*DMRT2*	c.887G>C	p.Ser296Thr	mis	exon4	VUS
60	0	0	ND	ND	ND	ND	ND	VUS
61	2	2	*HSD3B1*	c.1076C>G	p.Ser359Cys	mis	exon4	VUS
			*TSPO*	c.210G>C	p.Glu70Asp	mis	exon3	VUS
62	3	3	*CYP21A2*	c.1179C>G	p.His393Gln	mis	exon9	LP
			*LDB1*	c.770C>T	p.Ser257Leu	mis	exon9	VUS
			*GATA6*	c.203A>T	p.Glu68Val	mis	exon2	VUS
63	3	3	*GNG13*	c.166A>C	p.Asn56His	mis	exon3	VUS
			*CBX2*	c.565G>A	p.Ala189Thr	mis	exon5	VUS
			*CTNNB1*	c.395T>A	p.Leu132Gln	mis	exon4	VUS
64	0	0	ND	ND	ND	ND	ND	VUS
65	1	1	*TSPO*	c.440C>T	p.Thr147Met	mis	exon4	VUS
66	2	2	*POR*	c.160T>C	p.Phe54Leu	mis	exon2	VUS
			*PIK3AP1*	c.1730G>C	p.Arg577Thr	mis	exon11	VUS
67	1	1	*CAMK2D*	c.696 + 16T>G	ND	Splicing near	IVS9	VUS
68	2	2	*ADCY2*	c.211-3C>T	ND	Splicing near	IVS1	LB
			*DMRT1*	c.388G>A	p.Glu130Lys	mis	exon2	VUS
69	1	1	*SRD5A2*	c.196G>A	p.Gly66Arg	mis	exon1	VUS
72	1	1	*PIK3AP1*	c.13 + 12G>A	ND	Splicing near	IVS1	VUS
78	4	4	*LHX9*	c.716T>G	p.Ile239Ser	mis	exon3	VUS
			*NOBOX*	c.503A>G	p.His168Arg	mis	exon4	VUS
			*CYP11B1*	c.104T>C	p.Val35Ala	mis	exon1	VUS
			*ESR2*	c.688C>T	p.Arg230Trp	mis	exon5	VUS
84	4	4	*LHX9*	c.716T>G	p.Ile239Ser	mis	exon3	VUS
			*GLI2*	c.4571C>A	p.Ser1524Tyr	mis	exon14	VUS
			*TGFBI*	c.1312C>T	p.His438Tyr	mis	exon10	VUS
			*TRIM28*	c.839C>T	p.Ser280Leu	mis	exon5	VUS
86	2	2	*DMRT2*	c.535G>A	p.Gly179Arg	mis	exon3	VUS
			*POF1B*	c.862G>T	p.Asp288Tyr	mis	exon8	VUS

mis, missense variant; IVS, intron; ND, no available data; ACMG, American College of Medical Genetics and Genomics; LP, Likely pathogenic; VUS, Variants of uncertain significance; LB, Likely benign.

## 4 Discussion

Among known genetic contributors, *NR5A1* variants are among the most frequent monogenic causes of 46,XY DSD (1). *NR5A1* encodes SF-1, an “orphan” nuclear receptor (lacking a high-affinity ligand) that regulates transcription of genes essential for early sex determination (e.g., gonad formation) and differentiation. Patients with DSD carrying *NR5A1* variants show marked phenotypic variability without a consistent genotype–phenotype relationship. We analyzed the clinical, hormonal, and genetic characteristics of pediatric patients with *NR5A1* variants in a single-center Chinese cohort, focusing on genotype–phenotype correlations, intrafamilial variability, and potential oligogenic inheritance.

Notably, patients exhibited varying degrees of undermasculinization, ranging from female-appearing external genitalia with clitoromegaly to hypospadias with micropenis and/or cryptorchidism (22). At birth, common manifestations included atypical external genitalia with clitoromegaly, labial fusion, vaginal opening, or female genitalia, which may be misinterpreted as typical female anatomy, leading to female sex assignment. In our cohort, half of the patients were raised as female at birth, and sex reassignment from female to male occurred in 71% (32/45). Familial analysis showed that mothers carrying the same variants had POI or irregular menstruation, whereas fathers had DSD or oligospermia. These findings underscore the marked phenotypic heterogeneity and intrafamilial variability associated with identical variants.

Patients with *NR5A1* variants may present with Müllerian structures at birth. In our cohort, three patients with female-appearing or ambiguous external genitalia had Müllerian structures on imaging. Persistence of Müllerian remnants likely reflects impaired Sertoli cell function, as reduced AMH levels may result in incomplete Müllerian regression in *NR5A1* variants. PAI was observed in only three 46,XY patients, consistent with reports that PAI is uncommon in *NR5A1*-associated DSD. However, studies in heterozygous NR5A1+/− mice demonstrate adrenal insufficiency under stress with adrenal hyperplasia (23), suggesting that affected patients may have subclinical adrenal dysfunction that becomes clinically relevant during stress. Accordingly, long-term follow-up is warranted to assess age- or stress-related adrenal risk.

*NR5A1* is essential for splenic development, and variants have been associated with splenic anomalies (e.g., asplenia) in patients (13, 14, 24, 25) and mouse models (26). We did not assess this in our cohort because of limited data; however, such anomalies are likely underreported, as they are often detected only through targeted imaging. Given the increased risk of severe infections (e.g., sepsis and meningitis) associated with splenic dysfunction, screening for splenic anomalies in *NR5A1* variant carriers is recommended, as early detection may prevent life-threatening complications.

T concentrations were within the normal range in most patients, with evidence of mini-puberty or spontaneous puberty. Prepubertal patients generally showed an adequate T response to hCG stimulation, except for two individuals likely lacking functional Leydig cells. Elevated FSH concentrations and a reduced LH/FSH ratio, particularly during puberty, suggest that *NR5A1* variants preferentially impair Sertoli cell function. Consistently, AMH and inhibin B levels were lower in pubertal than prepubertal patients, indicating progressive Sertoli cell dysfunction with age, as previously reported (27). This decline may lead to oligozoospermia or azoospermia. Therefore, long-term monitoring of fertility potential and consideration of early fertility preservation are warranted in patients with *NR5A1*-related DSD.

Sex rearing in patients with DSD remains debated. Patients with *NR5A1* variants may undergo progressive masculinization if the gonads are retained. As shown in this study, some patients with *NR5A1* variants entered spontaneous puberty, and preserved fertility has also been reported (28). This is supported by the case of the father of six patients in our cohort, who carried the same variant as his affected child yet had preserved fertility. Collectively, these observations suggest potential advantages to rearing individuals with an *NR5A1* variant as boys, whereas rearing them as girls may be premature. Caution is warranted because early gonadectomy can cause irreversible consequences. Therefore, we suggest using gonadotropin-releasing hormone analogs to preserve gonadal function and suppress masculinization until the child reaches psychological maturity.

We identified 76 *NR5A1* variants in 87 individuals. Variants were distributed across the gene without apparent hotspots, and ~78% were non-recurrent. Exon 4 was most frequently affected, involving 32% (28/87) of patients. In addition, 37, 7 and 28 variants were located in the hinge region, LBD, and DBD, respectively, consistent with a previous SF-1 study (25). We identified 36 novel variants, expanding available variant datasets and informing disease pathogenesis. Recurrent changes clustered at amino acid positions 84 and 313 (p.Arg84His, n=3; p.Arg84Cys, n=2; p.Arg313Cys, n=4), suggesting these may represent relatively frequent variants in the Chinese population. Genotype–phenotype correlations remain unclear. We compared phenotypes by variant type (missense vs LoF) and by functional domain (DBD, LBD, and hinge region) and found no significant differences in EMS, testis location scores, PL-SDS, or sex assignment. Similarly, among recurrent variants, phenotypes differed even between individuals carrying the same *NR5A1* variant, arguing against a consistent genotype–phenotype relationship. In contrast, p.Arg92 variants are consistently associated with 46,XX (ovo)testicular DSD, potentially via downregulation of the pro-ovarian Wnt4/β-catenin pathway (29, 30). p.Gly35 variants are most commonly reported in both 46,XX and 46,XY individuals with PAI.

All individuals carried heterozygous *NR5A1* variants. Heterozygous inheritance is most commonly reported; however, the broad phenotypic spectrum is not readily explained by a dominant-negative mechanism (8). Haploinsufficiency has been proposed but remains unconfirmed (31). Multiple studies have interrogated the functional consequences of disease-causing *NR5A1* variants using *in vitro* assays and bioinformatic approaches (32–34); nevertheless, the basis for the weak genotype–phenotype correlation remains unclear. Additional hypotheses to explain the variability and complexity include: (a) tissue-specific somatic reversion, (b) additional genetic hits, (c) epigenetic regulation and other non-Mendelian contributions, and (d) gene–environment interactions. However, predicting how specific *NR5A1* variants alter SF-1 target gene expression and the abundance of interacting proteins, regulators, and modulators within adrenal and gonadal developmental and steroidogenic networks, and thereby shape the ultimate phenotype, remains challenging. In this study, we further examined the hypothesis that *NR5A1*-related DSD follow an oligogenic mode of inheritance. By reanalyzing WES data, we constructed a genetic map of potential oligogenic hits in patients with 46,XY DSD carrying heterozygous *NR5A1* variants, interpreted within current models of genetic interactions in adrenal and gonadal sex determination and development. The identified oligogenic candidates are not a random collection of genes; they converge on specific biological processes essential for sex development. For example, variants in steroidogenic enzymes (e.g., CYP21A2, HSD3B1) are expected to affect hormonal output, while variants in transcription factors (e.g., CBX2, DMRT1) may affect gonadal fate decisions. While the co-occurrence of these variants with NR5A1 mutations is statistically enriched, their exact contribution to the final phenotype likely reflects a cumulative ‘polygenic load’ that shifts the phenotypic threshold. We acknowledge that the current oligogenic analysis is primarily exploratory. The identified candidate variants are not proposed as independent causes of DSD, but rather as potential genetic modifiers that might explain the marked variability observed in NR5A1 carriers (e.g., one family member presenting with severe DSD while another shows only mild symptoms, with phenotypes not being entirely concordant). At the same time, the functional impact of these candidate variants requires further confirmation through molecular experiments or animal models. Nevertheless, our data provide supportive evidence for the oligogenic model previously proposed by Camats et al. (15), extending it to a Chinese population.

Despite being a large, single-center study, several limitations should be acknowledged. The sample size of each variant subgroup was relatively small, resulting in limited statistical power to identify subtle differences in phenotype. Standard comparative significance testing was used rather than formal equivalence testing, so non-significant *P* values do not confirm clinical equivalence between different groups. Owing to the institutional service scope, systematic adult reproductive assessments, including full gonadotropin and sex hormone profiling, semen analysis, and pelvic imaging, were unavailable for parental carriers. Parental phenotypic data were therefore acquired primarily via retrospective history collection, which may underestimate subclinical or mild parental phenotypes and constitute an inherent limitation of the present study. And although partial family data were available, comprehensive phenotypic and genetic information from all relatives could not be obtained. Moreover, many patients had not yet reached puberty; thus, long-term follow-up is required to determine final outcomes of gender assignment and gonadal function. Accordingly, our conclusions are based on currently available data and underscore the need for continued longitudinal follow-up of individuals with *NR5A1* variants into adulthood.

In conclusion, our findings confirm the broad clinical phenotypes and marked intrafamilial variability associated with identical variants. We emphasize the importance of targeted surveillance (e.g., adrenal function, splenic anomalies, and fertility potential) and tailored management, particularly in males, to optimize long-term outcomes. The novel variants identified expand the existing variant database and enhance understanding of DSD pathogenesis. Furthermore, our data provide additional support for an oligogenic contribution to *NR5A1*-related DSD, in which multiple variants in DSD-related genes may influence phenotypic expression.

## Data Availability

The original contributions presented in the study are included in the article/**Supplementary Material**. Further inquiries can be directed to the corresponding author.
