# Supplementary material for: NR5A1 gene variants: variable phenotypes, new variants, and genotype-phenotype correlations
Source: Front Endocrinol (Lausanne). 2026 Aug 13;17:1832270. doi: 10.3389/fendo.2026.1832270 (PMC13518173; doi:10.3389/fendo.2026.1832270)
Supplement: Supplementary file 1 [file Table1.docx]

Supplementary Material

# Supplementary Tables

**Supplementary Table 1. Clinical and genetic characteristics of NR5A1 variants in the Chinese single-center cohort.**

| **No.** | **Age**  **(yr)** | **Reared gender** | **EMS** | **LFS** | **UMS** | **PL-SDS** | **GLS**  **(L/R)** | **HGVS** | **Type** | **Domain** | **Source** | **ACMG** | **Repo-rted** | **Fam-ilial** |
| --- | --- | --- | --- | --- | --- | --- | --- | --- | --- | --- | --- | --- | --- | --- |
| 1 | 1.75 | M | 9 | 3 | 0 | -0.71 | 1.5/1.5 | NR5A1:NM_004959:exon4:c.338T>C:p.Ile113Thr | mis | / | / | VUS | NO |  |
| 2 | 0.29 | M | 0 | 0 | 0 | -3.86 | 0/0 | NR5A1:NM_004959:exon4:c.866T>C:p.Leu289Pro | mis | LBD | *de novo* | LP | NO |  |
| 3 | 2.91 | M | 9 | 3 | 0 | -0.33 | 1.5/1.5 | NR5A1:NM_004959:exon7:c.1315_1317delCAC:p.His439del | in-frame(del) | LBD | Father | LP | NO |  |
| 4 | 2.25 | F→M | 1 | 0 | 0 | -4.53 | 1/0 | NR5A1:NM_004959:exon2:c.86C>T:p.Thr29Met | mis | DBD | *de novo* | P | YES |  |
| 5 | 11 | F→M | 4 | 0 | 0 | -0.72 | 0.5/0.5 | NR5A1:NM_004959:exon7:c.1289G>T:p.Ser430Ile | mis | LBD | *de novo* | P | YES |  |
| 6 | 0.5 | M | 3 | 0 | 0 | -6.71 | 1.5/1.5 | NR5A1:NM_004959:exon4:c.245-2A>T | Lof(Splicing) | / | *de novo* | P | YES |  |
| 7 | 1.25 | F→M | 2 | 0 | 0 | -4.71 | 1/1 | NR5A1:NM_004959:exon4:c.645dupG:p.Pro216Alafs*10 | Lof (fs) | HR | Mother | LP | YES |  |
| 8 | 2.20 | F→M | 2.5 | 0 | 0 | -7.47 | 1.5/1 | NR5A1:NM_004959:exon4:c.259C>T:p.Arg87Cys | mis | DBD | *de novo* | LP | YES |  |
| 9 | 1.20 | F→F | 3 | 0 | 0 | / | 1.5/1.5 | NR5A1:NM_004959:exon7:c.1236C>A:p.Cys412* | Lof (non) | LBD | *de novo* | P | YES |  |
| 10 | 2 | F→F | 2 | 0 | 0 | -6.88 | 1/1 | NR5A1:NM_004959:exon2:c.63C>T:p.Ser21= | Syn | HR | Mother | VUS | NO |  |
| 11 | 6.58 | F→F | 2 | 0 | 0 | / | 1/1 | NR5A1:NM_004959:exon6:c.1138+1G>A | Lof(Splicing) | / | *de novo* | P | YES |  |
| 12 | 0.25 | M | 3 | 0 | 0 | -8.14 | 1.5/1.5 | NR5A1:NM_004959:exon4:c.614dupC:p.Gln206Thrfs*20 | Lof (fs) | HR | *de novo* | P | YES |  |
| 13 | 0.42 | F→M | 3 | 0 | 0 | -5.86 | 1.5/1.5 | NR5A1:NM_004959:exon4:c.250C>T:p.Arg84Cys | mis | DBD | Father | LP | YES |  |
| 14 | 8.75 | M | / | / | / | / | NA | NR5A1:NM_004959:exon4:c.259C>T:p.Arg87Cys | mis | DBD | NA | VUS | YES |  |
| **15** | 6.75 | F→M | 3 | 0 | 0 | -4.98 | 1.5/1.5 | NR5A1:NM_004959:exon5:c.982G>A:p.Gly328Arg | mis | LBD | Father | LP | YES |  |
| **16** | 10.50 | F→F | 3 | 0 | 0 | -2.36 | 0/0 | NR5A1:NM_004959:exon2:c.3G>A:p.Met1Ile | p.Met1? | / | Mother | LP | YES | + |
| **17** | 1.08 | F→M | 0 | 0 | 0 | -7 | 0/0 | NR5A1:NM_004959:exon4:c.247G>A:p.Val83Met | mis | DBD | *de novo* | LP | YES |  |
| **18** | 1.25 | M | 9 | 3 | 0 | -2.14 | 1.5/1.5 | NR5A1:NM_004959:exon5:c.937C>T:p.Arg313Cys | mis | LBD | Mother | LP | YES |  |
| **19** | 1.25 | M | 9 | 3 | 0 | -2.14 | 1.5/1.5 | NR5A1:NM_004959:exon4:c.251G>A:p.Arg84His | mis | DBD | Mother | LP | YES |  |
| **20** | 13.33 | M | / | / | / | / | / | NR5A1:NM_004959:exon4:c.756C>A:p.Thr252= | Syn | LBD | Father | VUS | YES | + |
| **21** | 16 | F→M | 0 | 0 | 0 | -1.73 | 0/0 | NR5A1:NM_004959:exon2:c.99C>A:p.Cys33* | Lof (non) | DBD | Mother | LP | YES | + |
| **22** | 0.92 | M | 6 | 3 | 0 | -3.86 | 1.5/1.5 | NR5A1:NM_004959:exon5:c.877G>A:p.Asp293Asn | mis | LBD | Mother | LP | YES |  |
| **23** | 1.16 | M | 4.5 | 0 | 0 | -2.43 | 1.5/0 | NR5A1:NM_004959:exon5:c.874_885delGCCGACCAGATG:p.Ala292_Met295del | in-frame(del) | LBD | Mother | VUS | NO |  |
| **24** | 0.42 | M | 9 | 3 | 0 | -3 | 1.5/1.5 | NR5A1:NM_004959:exon5:c.909C>A:p.Ser303Arg | mis | LBD | *de novo* | LP | YES |  |
| **25** | 0.50 | M | 1.5 | 0 | 0 | -8.14 | 0/1.5 | NR5A1:NM_004959:exon2:c.77G>A:p.Gly26Glu | mis | DBD | *de novo* | P | YES |  |
| **26** | 1.25 | F→M | 4 | 0 | 1 | -8.43 | 1.5/1.5 | NR5A1:NM_004959:exon7:c.1253A>C:p.Gln418Pro | mis | LBD | NA | VUS | NO |  |
| **27** | 12.66 | F→M | 5.5 | 0 | 0 | / | 1.5/1 | NR5A1:NM_004959:exon5:c.937C>T:p.Arg313Cys | mis | LBD | *de novo* | P | YES |  |
| **28** | 10.58 | F→F | 1 | 0 | 0 | / | 0/1 | NR5A1:NM_004959:exon4:c.274C>T:p.Arg92Trp | mis | DBD | *de novo* | P | YES |  |
| **29** | 0.83 | F→M | 3 | 0 | 0 | -4.71 | 1.5/1.5 | NR5A1:NM_004959:exon7:c.1273delG:p.Glu425Argfs*5 | Lof (fs) | LBD | *de novo* | P | YES |  |
| **30** | 1 | M | 3 | 0 | 0 | / | 1.5/1.5 | NR5A1:NM_004959:exon7:c.1289G>A:p.Ser430Asn | mis | LBD | *de novo* | LP | YES |  |
| **31** | 13.66 | M | / | / | / | / | / | NR5A1:NM_004959:exon5:c.990+20C>A | noncoding | / | Mother | VUS | NO |  |
| **32** | 1 | M | 9 | 3 | 0 | -1.85 | 1.5/1.5 | NR5A1:NM_004959:exon4:c.835T>G:p.Trp279Gly | mis | LBD | Mother | LP | NO |  |
| **33** | 0.16 | M | 3 | 0 | 0 | / | 1.5/1.5 | NR5A1:NM_004959:exon6:c.1048C>T:p.Arg350Trp | mis | LBD | Father | VUS | NO |  |
| **34** | 2 | F→M | 3 | 0 | 0 | -6 | 1.5/1.5 | NR5A1:NM_004959:exon6:c.991-9T>A | noncoding | / | Father | VUS | NO |  |
| **35** | 4.33 | M | 11.5 | 3 | 3 | -2 | 1.5/1 | NR5A1:NM_004959:exon3:c.185G>A:p.Arg62His | mis | DBD | Mother | LP | NO | + |
| **36** | 0.92 | F→M | 6 | 0 | 0 | -1.57 | 1.5/1.5 | NR5A1:NM_004959:exon3:c.193T>C:p.Cys65Arg | mis | DBD | Mother | LP | NO |  |
| **37** | 9.71 | M | 6 | 0 | 0 | -1.43 | 1.5/1.5 | NR5A1:NM_004959:exon5:c.937C>T:p.Arg313Cys | mis | LBD | *de novo* | P | YES |  |
| **38** | 0.25 | M | 6 | 3 | 0 | -3.86 | 1.5/1.5 | NR5A1:NM_004959:exon5:c.877G>A:p.Asp293Asn | mis | LBD | Mother | LP | YES | + |
| **39** | 6.08 | M | / | / | / | / | / | NR5A1:NM_004959:exon7:c.1365G>T:p.Met455Ile | mis | LBD | Father | VUS | NO |  |
| **40** | 0.83 | M | 3 | 0 | 0 | -3.86 | 1.5/1.5 | NR5A1:NM_004959:exon7:c.1256T>C:p.Leu419Pro | mis | LBD | Mother | VUS | NO |  |
| **41** | 0.17 | F→M | 3 | 0 | 0 | -6.71 | 1.5/1.5 | NR5A1:NM_004959:exon4:c.319C>T:p.Gln107* | Lof (non) | / | *de novo* | P | YES |  |
| **42** | 1.92 | F→M | 3 | 0 | 0 | -4.53 | 1.5/1.5 | NR5A1:NM_004959:exon3:c.194G>A:p.Cys65Tyr | mis | DBD | Father | LP | YES | + |
| **43** | 1 | F→M | 1 | 0 | 0 | -5.29 | 0/1 | NR5A1:NM_004959:exon7:c.1202T>C:p.Leu401Pro | mis | LBD | NA | VUS | NO |  |
| **44** | 2.75 | F→F | 0 | 0 | 0 | -5.12 | 0/0 | NR5A1:NM_004959:exon2:c.89G>A:p.Cys30Tyr | mis | DBD | Mother | LP | NO | + |
| **45** | 11 | F→M | 0 | 0 | 0 | -1.91 | 0/0 | NR5A1:NM_004959:exon5:c.938G>A:p.Arg313His | mis | LBD | *de novo* | P | YES |  |
| **46** | 6.17 | F→? | 3 | 0 | 0 | -3.81 | 1.5/1.5 | NR5A1:NM_004959:exon4:c.639C>A:p.Tyr213* | Lof (non) | HR | Father | VUS | NO | + |
| **47** | 1.5 | M | 6 | 0 | 0 | 0.14 | 1.5/1.5 | NR5A1:NM_004959:exon4:c.503C>A:p.Ala168Glu | mis | HR | Mother | VUS | YES |  |
| **48** | 0.92 | F→M | 1.5 | 0 | 0 | -4.43 | 0/1.5 | exon1-7 deletion | gross del | / | NA | LP | NO |  |
| **49** | 10 | F→M | 0 | 0 | 0 | -3.7 | 0/0 | NR5A1:NM_004959:exon6:c.1052C>A:p.Ala351Glu | mis | LBD | Mother | LP | YES | + |
| **50** | 11 | F→M | 2 | 0 | 0 | -2.95 | 1/1 | NR5A1:NM_004959:exon7:c.1250delA:p.Gln417Argfs*13 | Lof (fs) | LBD | Father | LP | YES |  |
| **51** | 0.58 | M | 0 | 0 | 0 | -3.86 | 0/0 | NR5A1:NM_004959:exon5:c.910_911delinsAG:p.Glu304Arg | in-frame (ins) | LBD | Father | VUS | NO | + |
| **52** | 0.5 | M | 9 | 3 | 0 | -2.14 | 1.5/1.5 | NR5A1:NM_004959:exon2:c.76G>A:p.Gly26Arg | mis | DBD | Mother | LP | YES |  |
| **53** | 0.42 | M | / | / | / | / | / | NR5A1:NM_004959:exon4:c.603T>A:p.Tyr201* | Lof (non) | HR | Mother | LP | YES |  |
| **54** | 2.33 | F→M | 0 | 0 | 0 | -3.06 | 0/0 | exon2-3 deletion | gross deletion | / | NA | LP | YES |  |
| **55** | 5.25 | M | / | / | / | / | / | NR5A1:NM_004959:exon4:c.262_263delAT:p.Met88Glufs*60 | Lof (fs) | DBD | Mother | LP | NO |  |
| **56** | 12.33 | F→M | / | / | / | / | / | NR5A1:NM_004959:exon6:c.1068_1076delGCAGCTGCT:p.Gln357_Leu359del | in-frame (del) | LBD | Father | VUS | NO | + |
| **57** | 0.75 | M | 2.5 | 0 | 1 | -8.14 | 1.5/0 | NR5A1:NM_004959:exon4:c.305_310delACCGGG:p.Asp102_Arg103del | in-frame (del) | DBD | Father | VUS | YES |  |
| **58** | 1.83 | F→M | 2 | 0 | 0 | -3.57 | 1/1 | NR5A1:NM_004959:exon6:c.1089_1090insCTTGCGCTGCAGCTG:p.Leu359_Leu363dup | in-frame (ins) | LBD | *de novo* | LP | YES |  |
| **59** | 1.5 | F→M | 3 | 0 | 1 | -3.29 | 1/1 | NR5A1:NM_004959:exon6:c.1048C>T:p.Arg350Trp | mis | LBD | Mother | VUS | NO |  |
| **60** | 4.75 | M | 8 | 3 | 3 | / | 1/1 | NR5A1:NM_004959:exon4:c.250C>T:p.Arg84Cys | mis | DBD | Mother | P | YES | + |
| **61** | 0.83 | M | 3 | 0 | 0 | -6.71 | 1.5/1.5 | NR5A1:NM_004959:exon4:c.251G>A:p.Arg84His | mis | DBD | Mother | LP | YES | + |
| **62** | 0.42 | M | 7 | 3 | 2 | -3.29 | 1/1 | NR5A1:NM_004959:exon3:c.119C>T:p.Thr40Met | mis | DBD | Father | LP | NO |  |
| **63** | 16.42 | M | / | / | / | / | / | NR5A1:NM_004959:exon6:c.1063G>A:p.Val355Met | mis | LBD | Mother | VUS | YES |  |
| **64** | 1 | M | 3 | 0 | 0 | -3.57 | 1.5/1.5 | NR5A1:NM_004959:exon7:c.1214C>A:p.Thr405Asn | mis | LBD | Father | VUS | NO |  |
| **65** | 5.83 | M | 6 | 3 | 0 | -3.78 | 1.5/1.5 | NR5A1:NM_004959:exon4:c.870+1G>A | Lof (Splicing) | / | *de novo* | P | NO |  |
| **66** | 1.5 | F→? | 2.5 | 0 | 0 | -2.71 | 1/1.5 | NR5A1:NM_004959:exon5:c.937C>T:p.Arg313Cys | mis | LBD | Mother | LP | YES |  |
| **67** | 1.16 | F→M | 2.5 | 0 | 0 | / | 1/1.5 | NR5A1:NM_004959:exon7:c.1183_1185del:p.Glu395del | in-frame (del) | LBD | Father | LP | YES |  |
| **68** | 5.83 | M | 11 | 3 | 2 | -2.39 | 1.5/1.5 | NR5A1:NM_004959:exon4:c.275G>A:p.Arg92Gln | mis | DBD | Father | P | YES |  |
| **69** | 0.92 | M | 5 | 3 | 0 | -3.29 | 1/1 | NR5A1:NM_004959:exon7:c.1367T>G:p.Leu456Arg | mis | LBD | Father | VUS | NO | + |
| **70** | 7.25 | F→F | 2 | 0 | 0 | -6.45 | 1/1 | exon1-3 deletion | gross del | / | *de novo* | P | NO |  |
| **71** | 0.58 | M | 3 | 0 | 0 | -6.14 | 1.5/1.5 | NR5A1:NM_004959:exon2:c.76G>T:p.Gly26* | Lof (non) | DBD | *de novo* | LP | NO |  |
| **72** | 5.58 | F→M | 5 | 0 | 0 | 0.11 | 1/1 | NR5A1:NM_004959:exon7:c.1139A>T:p.Asp380Val | mis | LBD | *de novo* | P | NO |  |
| **73** | 1.42 | F→M | 1.5 | 0 | 0 | -3.28 | 0/1.5 | NR5A1:NM_004959:exon5:c.990+1G>T | Lof(Splicing) | / | *de novo* | P | NO |  |
| **74** | 10.42 | M | / | / | / | / | / | NR5A1:NM_004959:exon4:c.634G>A:p.Gly212Ser | mis | HR | NA | LP | YES |  |
| **75** | 9.83 | F→M | 3 | 0 | 0 | -1.63 | 0/0 | NR5A1:NM_004959:exon6:c.1049_1069dup:p.Arg350_Leu356dup | in-frame (ins) | LBD | Mother | VUS | NO | + |
| **76** | 1.5 | F→M | 2 | 0 | 0 |  | 1.5/0.5 | NR5A1:NM_004959:exon4:c.251G>A:p.Arg84His | mis | DBD | *de novo* | P | YES |  |
| **77** | 1.25 | F→M | 1.5 | 0 | 0 | -4.14 | 0/1.5 | exon4,6 deletion | gross del | / | *de novo* | P | NO |  |
| **78** | 1.08 | F→M | 0 | 0 | 0 | -6.43 | 0/0 | NR5A1:NM_004959:exon3:c.104G>T:p.Gly35Val | mis | DBD | Father | P | YES |  |
| **79** | 0.83 | F | 3 | 0 | 0 | -4.71 | 1.5/1.5 | NR5A1:NM_004959:exon4:c.500del:p.Pro167Glnfs*129 | Lof (fs) | HR | *de novo* | P | NO |  |
| **80** | 9.92 | M | / | / | / | / | / | NR5A1:NM_004959:exon3:c.164G>A:p.Cys55Tyr | mis | DBD | NA | LP | YES |  |
| **81** | 11 | M | / | / | / | / | / | NR5A1:NM_004959:exon3:c.182A>C:p.Gln61Pro | mis | DBD | Mother | VUS | NO | + |
| **82** | 1.58 | F→M | 3 | 0 | 0 | -2.71 | 1.5/1.5 | NR5A1:NM_004959:exon4:c.614del:p.Pro205Hisfs*91 | Lof (fs) | LBD | *de novo* | P | NO |  |
| **83** | 1.5 | M | 3 | 0 | 0 | -7 | 1.5/1.5 | NR5A1:NM_004959:exon7:c.1236C>A:p.Cys412* | Lof (non) | LBD | Father | LP | YES | + |
| **84** | 11.16 | M | 9 | 3 | 0 | 0.03 | 1.5/1.5 | NR5A1:NM_004959:exon7:c.1139-17A>G | noncoding | / | Mother | VUS | NO |  |
| **85** | 0.16 | F→? | 2 | 0 | 0 | -5.29 | 1/1 | NR5A1:NM_004959:exon2:c.102G>C:p.Lys34Asn | mis | DBD | Mother | VUS | NO |  |
| **86** | 1.16 | F | 0 | 0 | 0 | -5.29 | 0/0 | NR5A1:NM_004959:exon4:c.275G>A:p.Arg92Gln | mis | DBD | *de novo* | P | YES |  |
| **87** | 0.16 | F | / | / | / | / | / | NR5A1:NM_004959:exon3:c.104G>A:p.Gly35Asp | mis | DBD | *de novo* | P | YES |  |

EMS, External masculinization scores; LFS, Labioscrotal fusion score; UMS, Urethral meatus score; PL-SDS, Penile length-standard deviation score; GLS, Gonads location score; L, Left; R, Right; HGVS, Human Genome Variation Society; ACMG, American College of Medical Genetics and Genomics; F, female; M, male; ?, undetermined; mis, missense; non, nonsense; fs, frameshift; dup, duplication; delins, deletion-insertion; ins, insertion; HR, Hinge Region; DBD, DNA binding domain; LBD, Ligand binding domain; P, Pathogenic; LP, Likely pathogenic; VUS, Variants of uncertain significance.
